# Surveillance of antimicrobial consumption: methodological review for systems development in Thailand

**DOI:** 10.7189/jogh.07.010307

**Published:** 2017-06

**Authors:** Viroj Tangcharoensathien, Angkana Sommanustweechai, Boonrat Chanthong, Nithima Sumpradit, Rungpetch Sakulbumrungsil, Sasi Jaroenpoj, Varavoot Sermsinsiri, on behalf of the Thai SAC Working Group

**Affiliations:** 1 International Health Policy Program, Ministry of Public Health, Nonthaburi, Thailand; 2 Faculty of Veterinary Science, Mahidol University, Nakornpathom, Thailand; 3 Food and Drug Administration, Ministry of Public Health, Nonthaburi, Thailand; 4 Faculty of Pharmaceutical Sciences, Chulalongkorn University, Bangkok, Thailand; 5 Department of Livestock Development, Ministry of Agriculture and Cooperatives, Phathumtani, Thailand

The increased trend of antimicrobial resistance has become a global threat to human security, causing serious negative impacts on human, animal and environment. Inappropriate uses of antimicrobial are main drivers of the emergence of resistant bacteria [1].Combating AMR requires in–country multi–sectoral actions and global collective efforts using “One Health” approach.

The Thai National Strategic Plan on AMR (2017–2021) was developed through a full engagement of stakeholders and National Health Assembly processes [2]. The Thailand Cabinet endorsed it in August 2016. Two out of the five targets are 20% and 30% reduction in antimicrobial consumption in human and animal by 2021, respectively (see **Box 1**).

Box 1 **Goals of the National Strategic Plan for AMR**

By 2021:

1. 50% reduction in AMR morbidity
2. 20% reduction in antimicrobial consumption in humans
3. 30% reduction in antimicrobial consumption in animals
4. 20% increase of public knowledge on AMR and awareness of appropriate use of antimicrobials
5. Capacity of the national AMR management system is improved to level 4 (measured by the 2016 WHO’s Joint External Evaluation for International Health Regulation 2005)

To optimize use of antimicrobial agents in human and animal, as recommended by the WHO Global Action Plan [3], countries need to develop a sustainable system which monitors their consumption and disseminate the information for policy decision. For example, France had a high human antibiotic use in the EU, and implemented a national campaign which resulted in a significant reduction in consumption [4]. This paper reviews international approaches on surveillance of antimicrobial consumption in human and animal, analyzes antimicrobial sales reporting systems and assesses how the surveillance system can be developed and sustained in Thailand.

## ANTIMICROBIAL CONSUMPTION IN HUMAN AND ANIMAL: REVIEW OF INTERNATIONAL APPROACHES

### Human consumption

Since 2011, European countries have been developing a surveillance system for human. The European Surveillance of Antimicrobial Consumption Network (ESAC–Net) [5], covers 30 European Union and European Economic Area (EU/EEA) countries. It provides cross–country analysis and information is fed back to member countries to inform policy, as well as making publicly assessable information through the interactive database.

Data sources are either national sales of antimicrobials or reimbursement data available from health insurance systems. These data disaggregate consumption by community (ambulatory care) and hospital care (**Table 1**).

**Table 1:** Two data sources used by ESAC–Net and ESVAC: reimbursement vs sales data

Country	Reimbursement data			Antimicrobial sales data			
Human, community uses	Human, hospital uses	Human, combined community and hospital uses	Human, community uses	Human, hospital uses	Human, combined community and hospital uses	Animal uses
Austria	X						X
Belgium	X	X					X
Bulgaria				X	X		X
Croatia*	X	X					
Cyprus			X				X
Czech Republic	X						X
Denmark				X	X		X
Estonia				X	X		X
Finland				X	X		X
France				X	X		X
Germany	X						X
Greece*				X	X		
Hungary	X						X
Iceland						X	X
Ireland		X		X	X		X
Italy		X		X			X
Latvia				X	X		X
Lithuania				X	X		X
Luxembourg	X				X		X
Malta*				X	X		
Netherlands				X	X		X
Norway	X	X		X	X		X
Poland				X			X
Portugal	X	X			X		X
Romania*			X				
Slovakia				X	X		X
Slovenia	X	X		X	X		X
Spain	X						X
Sweden				X	X		X
United Kingdom	X						X

*No data in animals.

The surveillance system may cover hospital or community levels [6]. Monitoring consumption in hospital settings is useful for impact assessment of AMR leading to attribution of morbidity and mortality on health care cost and for micro–policy decision on rational use. Monitoring consumption in community is more complex than a hospital setting, where national sales data to communities are used for estimation. A majority of developing countries do not compile national sales data to communities. Alternative ways of capturing antimicrobial use are surveys of pharmacies, sentinel in specific sites or prospective household survey.

### Animal consumption

The European Surveillance of Veterinary Antimicrobial Consumption (ESVAC) project was established in 2009 [7]. It reports antimicrobial consumption in animal by collecting sales data of veterinary antimicrobials in 26 countries, which covers 95% of total food–producing animal populations.

The data sources came from wholesalers (17 countries), marketing–authorization holders (4 countries), both wholesalers and marketing–authorization holders (2 countries). Some countries provide feed mill data (**Table 1**) [7]. In all countries, it is mandatory by Law for pharmaceutical operators to report their sales data to the national authority, except in France, Hungary, Netherlands and Spain.

### Analysis of data sources: reimbursement vs sales data

As seen in **Table 1**, antimicrobial sales data for human use is the main source in a majority of the 30 EU/EEA countries. These data are able to be disaggregated by community and hospital uses; while all countries are reliant on sales data for antimicrobial agents used in animals. Most European countries had achieved universal health coverage; still there are limitations in capturing antimicrobial consumption from reimbursement databases. In developing countries with limited population coverage by insurance schemes, more limitation of reimbursement data for estimate of antimicrobial consumption is expected.

This indicates that development of the surveillance system requires strengthening of antimicrobial sales data for both humans and animals. Reviews found that approaches used by EU/EAA countries can be applied to developing countries given there is a good antimicrobial sales data systems in both sectors.

## UNDERSTANDING THE LANDSCAPE OF DRUG AUTHORIZATION IN THAILAND

### Legal frameworks and actors

Two laws govern the distribution of antimicrobials for human and animals: the 1967 Drug Act responsible by Thai Food and Drug Authority (Thai–FDA); and the 2015 Animal Feed Quality Control Act, responsible by Department of Livestock Development. It is noted that a majority of antimicrobials used in livestock are consumed through medicated feeds, and much less on finished products mostly applied to pets.

Key actors are importers, local manufacturers and pharmacies which are authorized to sell antimicrobials by Thai–FDA. Antimicrobials can be used upon prescription, but in practice the requests from customers and farmers could influence dispensing in private pharmacies. A major loop–hole in enforcing the Drug Act is the lack of effective measures and monitoring systems for antibiotic distribution especially active pharmaceutical ingredients (API).

### Reporting sales data by operators

There are two mandatory sales reporting systems by the importers and manufacturers. First is the four–monthly report of sales and distribution of potentially abused medicines by consumers, such as steroids, tramadol and dextromethorphan. This system is designed for tight control of distribution of these medicines to prevent drug abuse. Second an annual report of production and importation of all pharmaceutical products where historically Thai–FDA did not request to provide distribution details.

**Figure 1:**
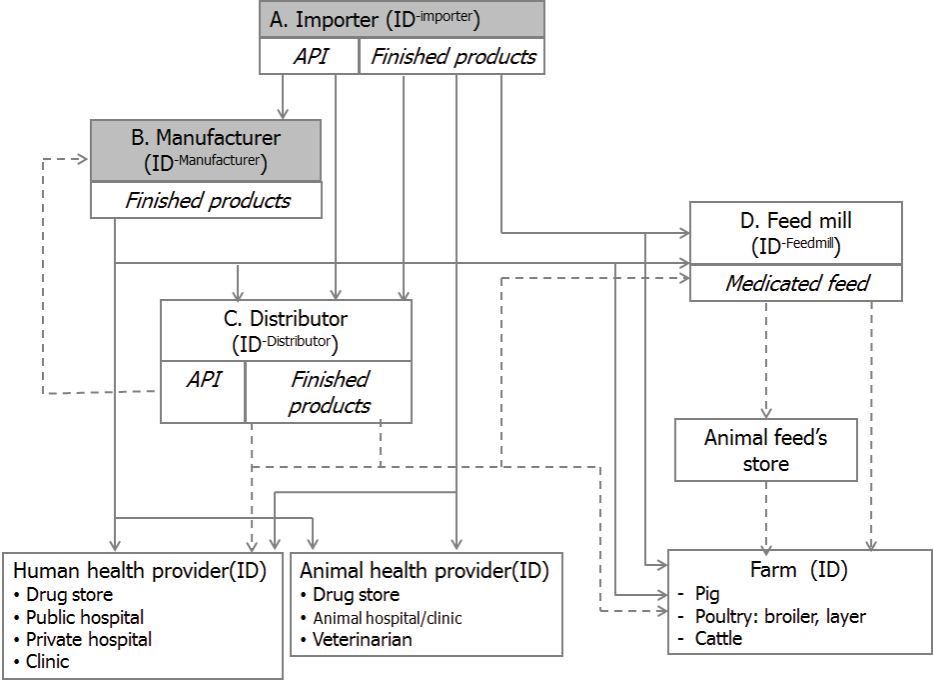
Distributional channels by antimicrobial agents. This study covers solid line which includes antibiotic sales data from importers and manufactures. The dotted line distributions are outside the scope of this study.

To facilitate development of surveillance system, the existing two reports needs to revise in order to track the distribution of antimicrobials from productions/importation to users. However, the Thai–FDA has to issue regulations to add highly potentially antibiotics to the four–monthly report. Moreover, a greater reliance on the annual report on production and importation with volumes and values of product and it requires them to report data on distribution channel. There is a gap which is the lack of reporting on the production of medicated feeds. **Figure 1** presents the distribution channels.

With reference to reviewed information from ESAC and ESVAC, the planned Thailand systems where data sources on total sales for human are the combine of community and hospital levels and sales data for animal happens to be similar to Iceland (**Table 1**) where antimicrobials sales data are available on combined community and hospital care in humans; and totals are available for animal use. The breakdown by community and hospital use will be discussed further in the subsequent section.

Key parameters in the electronic reporting will include: the operator’s unique ID; pharmaceutical product unique ID with reference to the License ID assigned by Thai–FDA; Anatomical Therapeutic Chemical (ATC) classification); quantity and value including package size; doses; and forms. The objectives are to estimate human consumption as measured by Defined Daily Dose (DDD) and animal consumption as measured by milligram of antibiotic.

## DEVELOPMENT OF SURVEILLANCE OF ANTIMICROBIAL CONSUMPTION: METHODOLOGICAL APPROACHES FOR THAILAND

Currently, there is no system to monitor consumption of antimicrobial in Thailand. The Thai Surveillance of Antimicrobial Consumption (Thai SAC) is developed to fill the gap, which provides a 2017 baseline consumption and a long term monitoring process. In 2017, grants from USAID, WHO and FAO had been approved for research and development of the Thai surveillance system. Research team comprises of Thai–FDA, Department of Livestock Development, universities and International Health Policy Program of the Ministry of Public Health. Systems analysis was conducted to understand the distributional channel, legal framework and sales reporting systems; all forms the basis of the surveillance system development.

All medicines in the Thai–FDA registration database are assigned with ATC classification code for human drugs and ATCvet for veterinary medicinal products. The scope of surveillance system will cover antimicrobials at least for systemic use, J01 in human and QJ01 in animal.

The design of Thai Surveillance of Antimicrobial Consumption is based on national sales data, which is a mandatory report by importers and manufacturers to the Thai–FDA; as use data by clinical conditions, age and gender are not readily available.

The antimicrobial sales data will be verified for their adequacy, accuracy and completeness before analysis. In human, antimicrobial consumption is measured by DDD per 1000 inhabitants–day [8]. The consumption of antimicrobials in animal is measured by milligrams of API per population correction unit (PCU). The PCU is the estimated weight for each animal species at treatment of livestock and of slaughtered animals at import and export for fattening and slaughter [7]. As there is no PCU in Thailand, we will use PCU following the ESVAC to facilitate international comparison.

**Figure:**
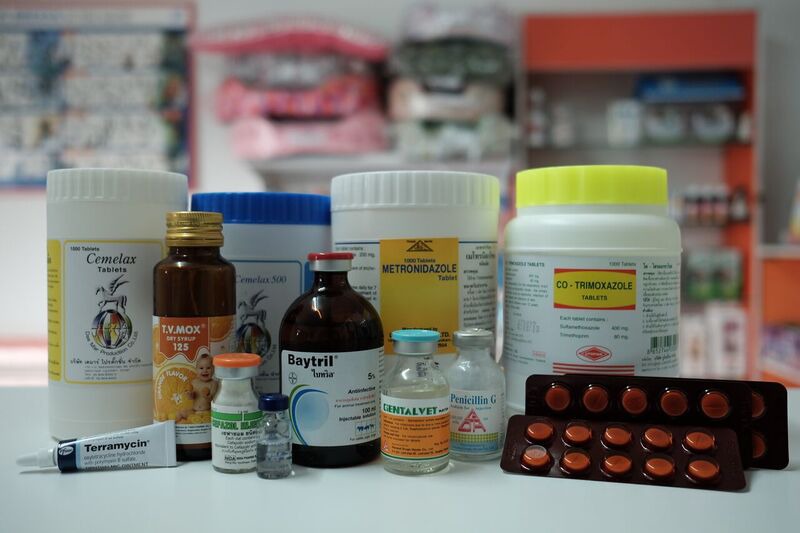
Photo: from the International Health Policy Program, Ministry of Public Health, Thailand, taken by Phanit Phacharaphimansakul (used with permission)

## CHALLENGES AND SOLUTIONS

A few challenges are identified for improvement of methodological approaches. Development of Surveillance of Antimicrobial Consumption relies on two sets of parameters: the numerators are total antimicrobial sales for human and animal consumption; the denominators are the total human and animal population.

### The numerators

The completeness and accuracy of reporting by operators, though mandatory, is an initial challenge. However, electronic submission with reference to the unique identification number of each ATC code would facilitate accuracy of reporting.

There are total 774 importers and 184 manufactures; for which representative samples of operators selected for on–site verification. This will gradually improve the quality of reports. Command and control, though a mandatory requirement by Thai–FDA, may not work well. Rather, effective communication between Thai–FDA and the operators and social recognition of their contributions to surveillance data are essential for adherence to quality report.

We assume that the total antimicrobial production and importation (though certain unknown size of reported illegal importation and production) minus total exports is the total consumption in both sectors. Although there is variation in annual stock, in an efficient pharmaceutical market, the stock level should be constant.

The annual report by operators does not disaggregate by community, hospital and animal species. For human antimicrobials, we plan to disaggregate by using national insurance reimbursement data set or surveys of organisations. Antimicrobial consumption by key species of food animals is important for specific policy intervention, efforts are planned to disaggregate data by special surveys, including the estimate of total consumption in the aquaculture.

The off–label use of human antibiotics in pets and plants in particular citrus trees for the treatment of Greening Diseases [9] is commonly observed in Thailand; efforts are under way to investigate sources and magnitude of antibiotic use in pets and plants with supports from FAO by this research program.

### The denominators

The Department of Livestock Development has yet to strengthen the data systems for accurate statistics on the total number of livestock by species. Not all livestock are raised through commercial standard farming systems, estimate size of local backyard farming contributes to accurate consumption per PCU. Estimate total national number of pets, most common are dogs and cats, and total PCU in aquaculture are the future research agenda.

### The sustainability of the surveillance system

Sustainability of the surveillance system not only depends on the mandatory reporting system, other enabling factors are for example effective communication with the operators, user friendly electronic submission, systems which facilitate e–submission and safeguard confidentiality of sales data.

Relevant authorities had fully involved in the surveillance system design and development; this ensures long term sustainability in particular the IT systems. Policy decision based on evidences and publicly accessible report foster political support for a sustainable Thai Surveillance of Antimicrobial Consumption.

## CONCLUSION

In responses to AMR, the Thai national surveillance system is critical for monitoring total consumption for which effective policies can be introduced to curb down excessive consumption. The current design disaggregates human consumption by level of care such as hospital and pharmacies; however point prevalence surveys are needed to estimate consumption by clinical conditions, age and gender. The current design does not differentiate consumption by animal species; further monitoring of veterinary prescription support consumption by animal species.

Reviews of international experiences and the analysis of how to design a Thai Surveillance of Antimicrobial Consumption are useful to developing countries to apply to suit the national data systems. The Political Declaration of the High–Level Meeting of the UNGA on AMR in September 2016 which calls for “Improve surveillance and monitoring of AMR and the use of antimicrobials to inform policies” [10] puts pressure on countries to develop surveillance system and ensure its use for policy decision.
